# Hepatitis B Research in Peru, 1988–2023: Geographic Inequities, Thematic Gaps, and Misalignment with Disease Burden

**DOI:** 10.3390/pathogens15070708

**Published:** 2026-07-06

**Authors:** Jhon Omar Palomino-Tenorio, Obert Marín-Sánchez, Jimmy Ango-Bedriñana, Ruy D. Chacón, Homero Ango-Aguilar

**Affiliations:** 1 Escuela Profesional de Biología, Programa de Estudios de Microbiología, Facultad de Ciencias Biológicas, Universidad Nacional de San Cristóbal de Huamanga (UNSCH), Ayacucho 05000, Peru; jhon.palomino.02@unsch.edu.pe; 2 Departamento Académico de Microbiología Médica, Facultad de Medicina, Universidad Nacional Mayor de San Marcos (UNMSM), Lima 15081, Peru; omarins@unmsm.edu.pe; 3 Pathogen Genetics Research Group (PATHO-GEN), Organization for Medical Innovation and Collaboration for Sciences (OMICS), Lima 15001, Peru; 4 Escuela Profesional de Medicina Humana, Facultad de Ciencias de la Salud, Universidad Nacional de San Cristóbal de Huamanga (UNSCH), Ayacucho 05000, Peru; jimmy.ango@unsch.edu.pe; 5 Department of Pathology, School of Veterinary Medicine, University of São Paulo, Sao Paulo 05508-270, Brazil

**Keywords:** hepatitis B virus, bibliometric analysis, scientometrics, epidemiology, research inequities, global health, Peru, public health surveillance

## Abstract

Hepatitis B virus (HBV) infection remains a major public-health challenge in Peru, particularly in historically hyperendemic Amazonian and Andean regions; however, the structure, evolution, and equity of national HBV research have not been systematically evaluated. We conducted a PRISMA-informed bibliometric analysis of all peer-reviewed and theses on HBV in Peru published between 1988 and 2023 using Scopus, Google Scholar, and the Peruvian National Repository (RENATI). Bibliometric indicators, collaboration networks, thematic structure, and temporal thematic evolution were analyzed in R using bibliometrix- and network-based approaches. The final corpus comprised 232 documents, with a marked increase in production after 2005 and a publication peak in 2018. Scientific output was strongly concentrated in Lima-based institutions, while several departments historically associated with HBV endemicity exhibited minimal or absent research production. Nearly half of the corpus corresponded to undergraduate and postgraduate theses. Thematic analyses revealed persistent predominance of epidemiology, seroprevalence, and vaccination-related research, whereas molecular virology, therapeutics, and translational research remained peripheral or poorly represented. International collaboration was markedly limited. Overall, Peruvian HBV research has expanded quantitatively but remains geographically centralized and shows only limited correspondence with the contemporary geographic distribution of HBV incidence, while also remaining only partially aligned with the contemporary global HBV research frontier. These findings provide an evidence-based framework to guide research-priority setting, territorial equity policies, and strategic investment in infectious disease research capacity in Peru. Moreover, the weak association observed between scientific production and departmental HBV incidence suggests that factors beyond contemporary epidemiological burden contribute to the current distribution of research activity in Peru, highlighting a critical but often overlooked dimension of health inequity in low- and middle-income countries (LMIC) research systems.

## 1. Introduction

Chronic infection with the hepatitis B virus (HBV) remains one of the most prevalent bloodborne viral infections worldwide and a leading cause of cirrhosis and hepatocellular carcinoma. The World Health Organization, in its most recent global hepatitis report, has reaffirmed the elimination of viral hepatitis as a public-health priority for 2030, conditional on sustained scale-up of universal infant vaccination, comprehensive blood-donor screening, expanded antiviral therapy and rigorous epidemiological surveillance, particularly in low- and middle-income countries (LMICs) where access gaps persist [1]. The biology, natural history and therapeutic landscape of chronic HBV infection have been comprehensively reviewed elsewhere [2]; here we focus on the research infrastructure that underpins national progress toward these targets.

Within Latin America, HBV epidemiology is characterized by substantial heterogeneity in genotype distribution, transmission dynamics, endemicity patterns and surveillance capacity across countries and subregions. Recent molecular epidemiological studies conducted throughout the region have highlighted the complex circulation, diversification and dissemination of multiple HBV genotypes associated with distinct historical, demographic and migratory processes [3,4,5,6]. Peru represents a particularly relevant setting for HBV research because historically hyperendemic transmission has been documented in several Amazonian and Andean regions, including periods of high prevalence and intense horizontal transmission in early life prior to widespread vaccination efforts [7,8,9,10]. Marked geographic heterogeneity has also been reported, with HBsAg prevalence ranging from approximately 1–3.5% in coastal populations to 9.8% in Ayacucho and up to 20% in some Amazonian Indigenous communities and hyperendemic localities such as Huanta before large-scale vaccination programs were implemented [10]. These epidemiological patterns contributed substantially to the burden of chronic liver disease and hepatocellular carcinoma in endemic communities. In Peru, HBV vaccination was first deployed through pilot programs in hyperendemic regions during the early 1990s, subsequently expanded to moderate- and high-endemicity areas in 1996, and finally incorporated into nationwide universal infant immunization in 2003 through the Expanded Program on Immunization (EPI); and later expanded to nationwide infant coverage; current clinical and prevention guidance for HBV in the country is consolidated in a national technical norm issued by the Ministry of Health [10,11]. Despite these structural commitments, evidence of persistent transmission has been documented in specific endemic settings—for example, among household contacts of chronic HBV carriers in the highland province of Huanta (Ayacucho) [12]—and a recent comprehensive review of pre- and post-vaccination seroprevalence in Ayacucho has summarized the available evidence for that region [10].

Bibliometric analysis offers a structured framework to characterize the production, geography, authorship and thematic evolution of a research field, generating actionable evidence about where scientific capacity is concentrated and where structural gaps persist [13,14]. In Peru, the scientific record on HBV combines indexed peer-reviewed literature with a substantial body of academic theses, the publication volume of which was reshaped by the 2014 Peruvian University Law (Law 30220 [15]) that established a defended thesis as a graduation requirement [16]. To our knowledge, no comprehensive bibliometric account of HBV research in Peru has been published; this absence limits the ability of policy makers, funding agencies and research institutions to align scientific priorities with epidemiological need.

Here we present a PRISMA-informed bibliometric analysis of all peer-reviewed and gray-literature documents addressing HBV in Peru and published between 1988 and 2023, integrating three complementary sources: Scopus, Google Scholar and the Peruvian National Repository (RENATI). We hypothesized that scientific output in this field would exhibit (i) marked concentration in a small number of institutions based in the national capital, (ii) under-representation of regions with documented epidemiological burden, and (iii) thematic concentration in descriptive epidemiology relative to current frontiers in HBV virology and therapeutics. Our objective was to quantify the alignment between Peruvian scientific production and the country’s HBV burden over five decades, and to provide a robust baseline for the next cycle of national research priorities.

## 2. Materials and Methods

### 2.1. Study Design and Reporting Standards

We conducted a bibliometric retrospective study of all peer-reviewed and Peruvian theses addressing HBV infection in Peru, published between 1988 and 2023. Reporting followed the PRISMA-informed statement [17], adapted to a bibliometric survey rather than a systematic review of clinical effectiveness.

### 2.2. Data Sources and Search Strategy

Three complementary information sources were queried in December 2023 to maximize both indexed and theses literature coverage: (i) Scopus (Elsevier) for peer-reviewed mainstream science; (ii) Google Scholar for bilingual coverage of regional and non-indexed outlets; and (iii) RENATI (the Peruvian National Repository of Research Works), to capture undergraduate and postgraduate theses (https://renati.sunedu.gob.pe/, accessed on 18 May 2026). Theses were intentionally included because they represent a substantial component of scientific production in Peru and other low- and middle-income countries (LMICs), where a considerable proportion of research output is disseminated through institutional repositories rather than indexed journals. The search syntax combined HBV-related descriptors with Peru-related terms using Boolean operators. No language restrictions were applied beyond English and Spanish. In Scopus, the query was applied to title, abstract, and keywords: TITLE-ABS-KEY ((“Hepatitis B” OR “HBV” OR “VHB”) AND (“Peru” OR “Perú”)). In Google Scholar, the query was applied to title: (intitle: “Hepatitis B” OR intitle: “HBV” OR intitle: “VHB”) AND (“Perú” OR “Peru”). In RENATI, after applying manual filters to limit the search to undergraduate and graduate theses, the query was applied to the title: “Hepatitis B”.

The Google Scholar search was conducted through the platform web interface on 13 February 2026. A title-restricted strategy (intitle:) was adopted to maximize thematic specificity and prioritize documents in which hepatitis B represented the primary focus of the study. An exploratory sensitivity search performed without the intitle restriction retrieved approximately 17,500 records, indicating substantially lower thematic specificity. Therefore, the title-restricted strategy was retained for corpus construction.

Records retrieved from all sources were consolidated into a single database. Deduplication was performed using title, authorship, publication year, and source information, applying a hierarchical retention rule prioritizing Scopus over RENATI over Google Scholar. Subsequently, records underwent manual metadata verification to identify residual duplicates, indexing inconsistencies, incomplete entries, and bibliographic errors before final corpus construction.

### 2.3. Eligibility Criteria

Considering the filters applied, the eligibility criteria were as follows:

- Inclusion criteria: original research, reviews, and theses published between 1988 and 2023; addressing HBV infection in human populations within Peruvian territory; written in English or Spanish; available in full text or with sufficient metadata.
- Exclusion criteria: conference abstracts, editorials, technical reports, letters to the editor, panels; studies on non-human HBV models; and studies whose data collection occurred entirely outside Peru.

### 2.4. Study Selection and PRISMA Flow

The database search identified 1403 records, including 181 from Scopus, 1000 from Google Scholar, and 222 from RENATI. After deduplication (*n* = 218 removed using a hierarchical retention rule prioritizing Scopus over RENATI over Google Scholar), 1185 records underwent title-and-abstract screening, of which 933 were excluded (767 by geography, 153 by topic, and 13 by document type). A total of 252 reports were sought for retrieval; 17 could not be obtained and 7 were excluded after full-text assessment, resulting in 228 eligible documents from database sources.

In parallel, citation snowballing identified 6 additional records. Of these, 2 could not be retrieved and 4 met the eligibility criteria.

The final bibliometric corpus therefore comprised 232 documents, including 228 identified through database searching and 4 identified through citation snowballing (Figure S1).

Title-and-abstract screening was initially conducted by one investigator (J.O.P.-T.). Full-text eligibility assessment and all uncertain inclusion decisions were subsequently reviewed with a senior investigator (H.A.-A.) to ensure consistency with the predefined eligibility criteria. Because duplicate independent screening was not performed throughout all selection stages, formal inter-reviewer agreement statistics were not calculated.

### 2.5. Data Extraction, Normalization, and Bibliometric Analysis

A purpose-built data extraction matrix was developed in Microsoft Excel to systematize bibliographic and methodological information from the retrieved records. Author names and institutional affiliations were manually standardized to reduce inconsistencies across records and improve analytical coherence. The original Spanish thematic classification used in the curated database was translated into English biomedical terminology to facilitate international interpretation and thematic aggregation; the translated categories are provided in Supplementary Tables.

Bibliometric indicators followed standard descriptive approaches commonly applied in scientometric studies, including annual scientific production, productivity per author, document-type distributions, study-design distributions, and author productivity patterns according to Lotka’s law. Co-authorship and institutional collaboration networks were constructed to explore scientific collaboration patterns. Thematic structure and temporal evolution of research topics were evaluated through thematic clustering and longitudinal topic analysis across different time periods. Thematic descriptors were extracted from the included records and manually normalized through synonym harmonization and thematic standardization to reduce redundancy and improve conceptual consistency. Co-occurrence networks were constructed using keywords occurring in at least two documents, and thematic communities were identified using the Louvain modularity algorithm. Geographic distribution of scientific production within Peru was assessed using choropleth mapping and descriptive coverage analysis, while international collaboration patterns were visualized through global collaboration mapping. Geographic attribution was based on the department where data collection, sample acquisition, or fieldwork was conducted. For multicenter studies, all explicitly reported departments were recorded. Institutional affiliation and corresponding-author address were not used for geographic assignment. To evaluate the relationship between scientific production and epidemiological burden, a Spearman rank correlation analysis was performed between the number of publications assigned to each department and the corresponding HBV incidence rate reported in 2023.

Author productivity followed Lotka’s inverse-power law; the exponent was estimated by ordinary least squares on the log–log distribution of authors versus papers (*n* = 2.06, R^2^ = 0.84). Because authors are clustered through co-authorship, thereby violating the independence assumption underlying conventional confidence intervals, no parametric confidence interval is reported. Instead, a cluster-robust bootstrap based on document-level resampling (B = 2000) was performed as a sensitivity analysis and yielded a consistent estimate, supporting the stability of the exponent.

To explore the relationship between scientific production and contemporary epidemiological burden, a geographic correlation analysis was performed at the departmental level. HBV case counts for 2023 were obtained from the Peruvian Epidemiological Bulletin (Vol. 32, Epidemiological Week 52 from: https://www.dge.gob.pe/portalnuevo/informacion-publica/boletines-epidemiologicos/, accessed on 18 May 2026), while population estimates were retrieved from the National Health Information Repository (REUNIS, https://www.minsa.gob.pe/reunis/), based on official projections from the National Institute of Statistics and Informatics (INEI, https://www.gob.pe/inei/). Department-specific HBV incidence rates were calculated per 10,000 inhabitants. Scientific production was quantified as the number of publications assigned to each department according to the reported study location (i.e., where data collection, sampling, or fieldwork was conducted), regardless of author affiliation. The association between HBV incidence and scientific output was evaluated using Spearman’s rank correlation coefficient (ρ). Data analysis and visualization were performed in RStudio (version 4.5.3) using the packages *ggplot2*, *ggpubr*, and *ggrepel*. A scatter plot incorporating median reference lines and a locally estimated scatterplot smoothing (LOESS) curve was generated to facilitate interpretation of spatial patterns.

### 2.6. Software and Reproducibility

All analyses and graphical visualizations were performed in RStudio (R Foundation for Statistical Computing, Vienna, Austria) using bibliometric, network-analysis, and data visualization packages, including bibliometrix, igraph, ggplot2, ggalluvial, and related dependencies. The analytical workflow, figures, and Supplementary Materials were organized through reproducible R-based scripts developed for this study. All scripts required to reproduce the analyses and figures are publicly available at: https://github.com/jhonpalomino02-blip/HBV-Bibliometrics-Peru/tree/main, accessed on 18 May 2026.

## 3. Results

### 3.1. Temporal and Geographic Distribution of Scientific Production

**Figure 1:**
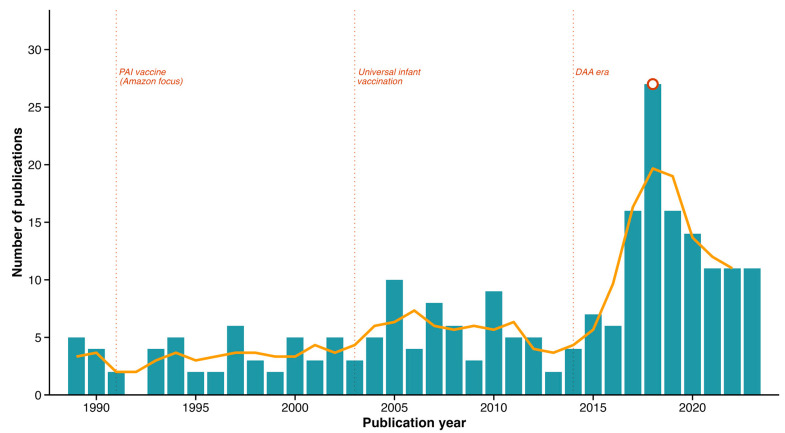
Historical trend of hepatitis B research output in Peru, 1988–2023. Bars represent the annual number of publications, while the solid orange line indicates the 3-year moving average of research output (*n* = 232 documents analyzed). Vertical dotted lines denote key epidemiological and public health landmarks in Peru, including the implementation of the Expanded Program on Immunization (EPI) vaccine focused on the Amazon region (1991), the introduction of universal infant vaccination (2003), and the onset of the direct-acting antiviral (DAA) era (2014). The red circle highlights the peak in annual publications observed in 2018.

The final bibliometric corpus comprised 232 documents published between 1988 and 2023, reflecting the progressive consolidation of hepatitis B research in Peru over recent years (Figure 1). Scientific production remained limited during the 1990s, followed by sustained growth after 2005 and a marked increase between 2015 and 2022. This temporal expansion coincided with the implementation of vaccination programs, the incorporation of molecular diagnostic techniques, and increased institutional participation in infectious disease research.

**Figure 2:**
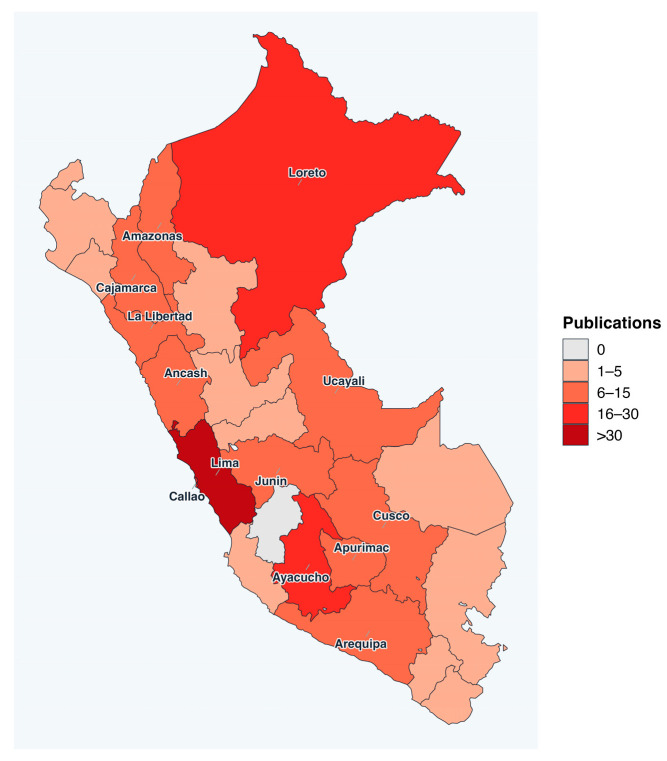
Spatial distribution of hepatitis B research output across Peruvian departments. The choropleth map illustrates the cumulative volume of HBV-related publications per geographic department (*n* = 232 documents analyzed). Publications were assigned to departments based on the reported location of data collection, not on author affiliation. Color density scales correspond to the frequency of scientific production, with specific key areas highlighted to emphasize regional disparities.

Geographic distribution analysis demonstrated a strong concentration of scientific production in Lima, which accounted for most publications included in the dataset. In contrast, several Amazonian and Andean departments historically associated with high hepatitis B endemicity showed comparatively low research output. Choropleth mapping revealed substantial territorial asymmetries in scientific productivity, highlighting persistent regional disparities in research infrastructure and scientific representation (Figure 2). Despite the overall increase in publication frequency over time, research activity remained unevenly distributed across both geographic regions and institutional centers.

**Figure 3:**
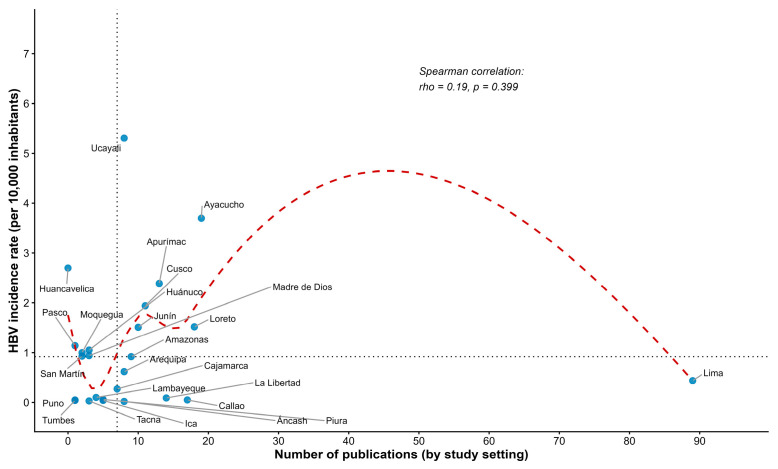
Association between departmental HBV incidence and scientific production in Peru. Each point represents a Peruvian department. The *x*-axis shows the number of HBV-related publications assigned according to study location, and the *y*-axis shows the HBV incidence rate in 2023 (per 10,000 inhabitants). Dotted lines indicate median values, and the red dashed line represents a LOESS curve.

To evaluate the relationship between scientific production and epidemiological burden, we assessed the association between departmental publication output and HBV incidence in 2023. Spearman correlation analysis revealed a weak and non-significant association between the number of publications and incidence rates across departments (ρ = 0.19, *p* = 0.399), suggesting that contemporary research activity is not strongly aligned with the current geographic distribution of HBV burden in Peru (Figure 3).

### 3.2. Document Types, Study Designs, and Research Approaches

Original articles represented the predominant document type within the analyzed corpus, followed by undergraduate and postgraduate theses, and narrative reviews, with Spanish being the most frequent publication language (Figure S2, Supplementary Tables). Descriptive and observational designs accounted for most studies, whereas analytical, longitudinal, and experimental approaches were comparatively less frequent. Public health and epidemiological investigations predominated throughout the study period, reflecting the historical emphasis of hepatitis B research in Peru on surveillance, seroprevalence, and endemic transmission.

**Figure 4:**
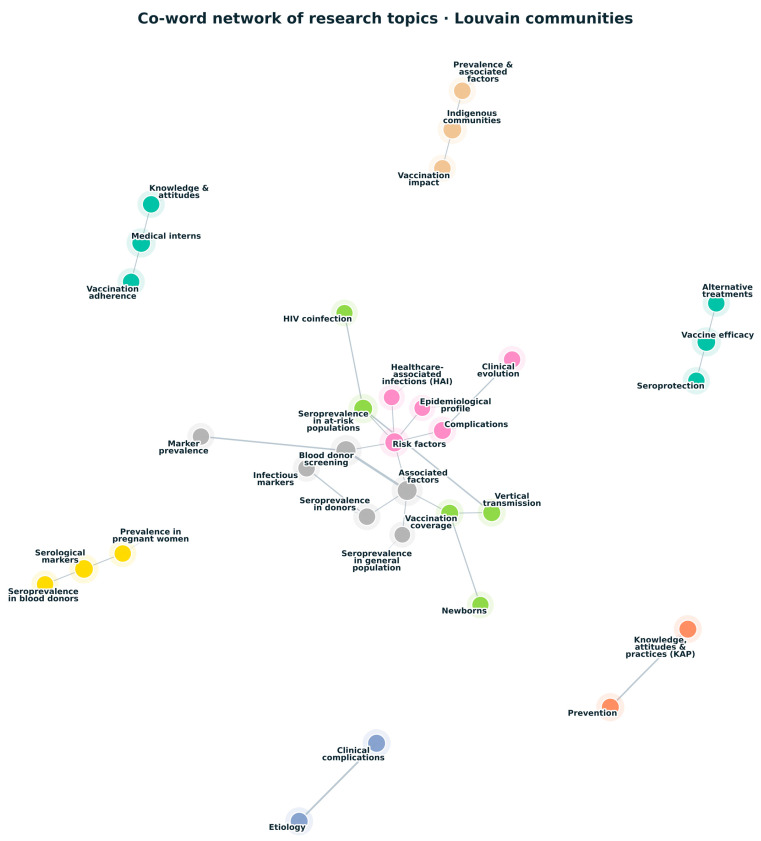
Co-word network of thematic concepts in Peruvian hepatitis B research. The network was constructed using normalized keywords occurring in at least two documents and includes 32 concepts connected by 28 co-occurrence edges. Nodes represent thematic concepts, with colors indicating communities detected through Louvain modularity. The network reveals both dominant and peripheral research themes within the Peruvian HBV literature.

Thematic mapping identified three principal research clusters within the scientific corpus (Figure 4). “Epidemiology & Public Health” constituted the dominant motor theme, showing the highest thematic centrality and density within the strategic diagram. “Prevention & Control” represented a highly developed but comparatively more specialized thematic area, whereas “Clinical & Therapeutic” topics occupied a peripheral and transversal position characterized by lower thematic cohesion and reduced interaction with other research domains. Overall, the thematic structure revealed a strong predominance of public health-oriented research with comparatively limited consolidation of clinical and therapeutic investigations. The revised network, constructed using keywords occurring in at least two documents, comprised 32 thematic concepts organized into nine thematic communities, providing a broader representation of the research landscape while preserving the same dominant thematic structure.

To further explore low-frequency and peripheral research topics, all normalized thematic descriptors were additionally visualized using a thematic treemap (Figure S3). This analysis revealed a broader thematic spectrum spanning Epidemiology & Public Health, Prevention & Control, Clinical & Therapeutic research, Basic Sciences & Virology, and Management & Policy, while confirming the predominance of epidemiological and public health themes within the overall corpus.

### 3.3. Author Productivity and Collaboration Networks

**Figure 5:**
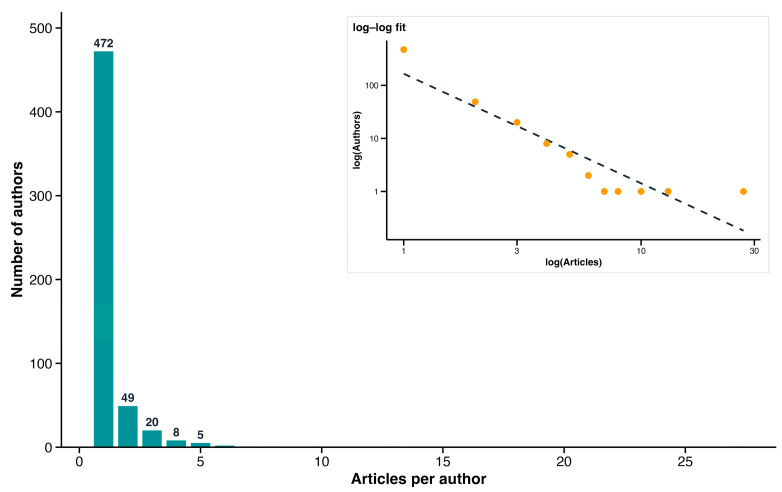
Distribution of author productivity in Peruvian hepatitis B research according to Lotka’s Law. Distribution of the number of publications per author within the HBV research corpus analyzed. The main panel shows the observed frequency of authors by publication count. The inset displays the log–log relationship between the number of publications and the corresponding number of authors, together with the fitted regression line used to estimate the Lotka exponent. Author productivity followed an inverse-power distribution with an estimated Lotka exponent of *n* = 2.06 (R^2^ = 0.84), indicating a strong concentration of scientific production among a small proportion of authors.

Author productivity analysis demonstrated a markedly asymmetric distribution consistent with Lotka’s law, in which a small number of researchers accounted for a substantial proportion of the total scientific production (Figure 5). Most authors contributed to only one publication, whereas a limited group maintained sustained productivity across multiple years.

**Figure 6:**
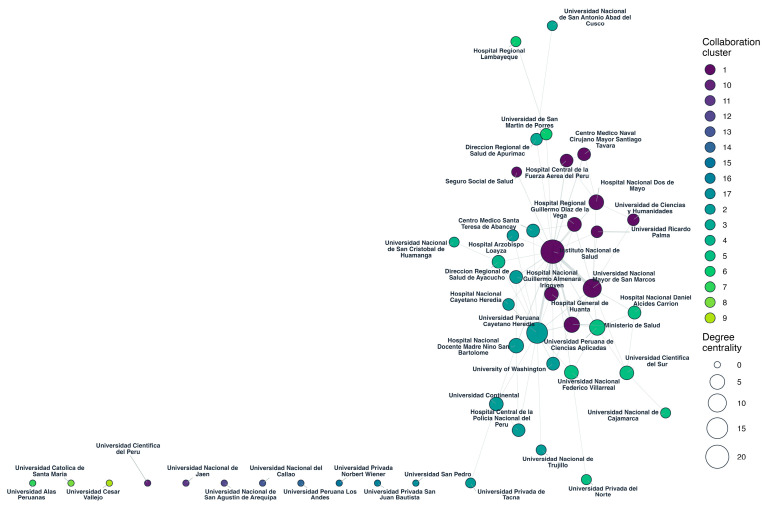
Institutional collaboration network in Peruvian hepatitis B research. The network diagram illustrates co-authorship interconnections among domestic and international institutions (*n* = 45 institutions with ≥2 documents, connected by *n* = 66 edges). Nodes represent individual institutions, with node size proportional to degree centrality, indicating the total number of collaborative links. Colors represent distinct institutional communities or clusters detected via Louvain modularity (17 total clusters), distinguishing central co-authorship hubs from peripheral or isolated institutional partnerships.

Co-authorship network analysis revealed the presence of relatively consolidated collaborative clusters centered around national public health institutions and major universities (Figure 6). Institutional collaboration networks showed strong participation by the Instituto Nacional de Salud, Universidad Nacional Mayor de San Marcos, and other Lima-based academic centers. International collaboration remained markedly limited, although cooperative links with institutions from the United States, Brazil, and Europe became more visible during recent years (Figures S4 and S5). Global collaboration mapping indicated that international partnerships were concentrated in molecular epidemiology, indigenous population studies, and viral surveillance initiatives.

### 3.4. Thematic Structure and Evolution of Hepatitis B Research

**Figure 7:**
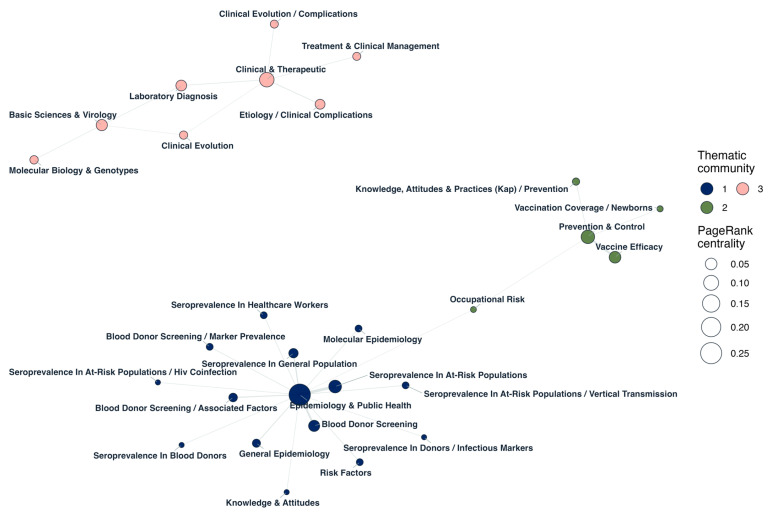
Thematic structure network of Peruvian hepatitis B research. The network includes 28 thematic concepts connected by 27 co-occurrence edges and grouped into three major thematic communities identified using Louvain modularity. Node size is proportional to PageRank centrality, reflecting the relative prominence of each thematic concept within the overall research landscape.

Co-occurrence network analysis based on keywords occurring in at least two documents identified three major thematic communities within hepatitis B research in Peru (Figure 7). The network comprised 28 thematic concepts connected by 27 co-occurrence links. The largest and most interconnected cluster was centered on “Epidemiology & Public Health”, incorporating topics related to seroprevalence, blood donor screening, risk factors, transmission dynamics, and at-risk populations. This thematic community occupied the most central position within the network and exhibited the highest PageRank centrality, indicating its dominant role in structuring the scientific corpus. Additional thematic clusters were associated with “Prevention & Control”, including vaccine efficacy and prevention-related studies, and with “Clinical & Therapeutic” research, which encompassed laboratory diagnosis, virology, and clinical complications.

**Figure 8:**
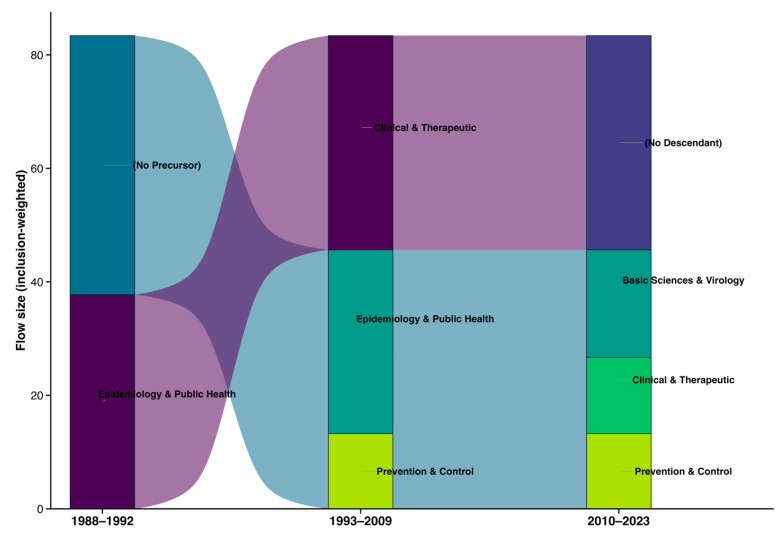
Thematic evolution of Peruvian hepatitis B research across three historical periods (1988–2023). The Sankey alluvial diagram illustrates the temporal shift, split, and convergence of research themes across three equal-length chronological phases. Vertical blocks represent detected thematic clusters within each period via Louvain modularity on co-keyword networks, where block height reflects flow size based on inclusion-weighted metrics. Stream fields (alluvial flows) trace the structural persistence and transformation of topics between consecutive periods, filtered by an Inclusion Index threshold of ≥0.20 to distinguish continuous thematic lineages from emerging concepts with no precursors or declining topics with no descendants.

Temporal thematic evolution demonstrated the sustained predominance of epidemiological and public health-oriented research across all historical periods analyzed (Figure 8). During 1988–1992, scientific production was largely concentrated in epidemiology and public health themes, with limited thematic diversification. Between 1993 and 2009, “Prevention & Control” and “Clinical & Therapeutic” topics emerged more prominently, indicating gradual expansion of the research landscape. In the most recent period (2010–2023), themes related to “Basic Sciences & Virology” became increasingly visible, while epidemiology and public health remained the principal thematic axis throughout the entire timeline. The alluvial structure additionally revealed partial thematic discontinuities, evidenced by clusters lacking clear precursors or descendants across successive periods.

## 4. Discussion

This study provides the first comprehensive overview of hepatitis B virus (HBV) research in Peru across more than three decades and reveals a striking dissociation between scientific production, epidemiological burden, and the contemporary global research frontier. Although HBV-related output expanded substantially after 2005 and peaked in 2018, this growth occurred within a highly centralized and thematically restricted research ecosystem dominated by Lima-based institutions and descriptive epidemiological studies. The findings therefore support all three initial hypotheses: scientific productivity was geographically concentrated, endemic regions remained underrepresented, and thematic development lagged behind the international transition toward molecular virology, immunopathogenesis, and therapeutic innovation.

The temporal growth pattern observed in this study is consistent with broader trends reported across Latin American biomedical research, where expansion in publication volume has often been driven more by structural educational reforms than by sustained increases in competitive research capacity [18,19,20,21,22]. In Peru, the implementation of University Law 30220 in 2014 introduced the defended thesis as a mandatory graduation requirement, substantially increasing academic production, particularly in undergraduate and postgraduate students [16]. Similar effects have been documented in Peruvian medical schools, where institutional licensing reforms and thesis requirements were associated with abrupt increases in scientific output but not necessarily with proportional improvements in international visibility or long-term research infrastructure [23]. The marked 2018 publication peak observed in our corpus likely reflects this phenomenon. However, nearly half of the retrieved documents corresponded to theses, suggesting that volumetric growth alone may overestimate the consolidation of sustainable scientific ecosystems. This distinction is particularly relevant in LMIC contexts, where bibliometric expansion may coexist with persistent fragility in funding, mentorship, and laboratory infrastructure [18,22].

One of the most consequential findings of this study is the pronounced geographic asymmetry in HBV research production within Peru. Lima concentrated the majority of publications despite historically exhibiting lower HBV endemicity than Amazonian and Andean regions such as Loreto, Ucayali, Madre de Dios, and Ayacucho [7,9,10,24]. Even more strikingly, Huancavelica produced no indexed publications across the entire study period despite longstanding evidence of viral hepatitis transmission in highland populations. This “production-burden paradox” mirrors patterns described throughout Latin America, where scientific production tends to cluster around metropolitan universities and national research institutes regardless of disease geography [22,25,26]. This interpretation is further supported by the absence of a significant association between departmental publication output and contemporary HBV incidence. Although some endemic regions generated substantial research activity, overall scientific production did not closely track the current geographic distribution of disease burden. These findings should be interpreted cautiously because research priorities are also influenced by historical endemicity, institutional capacity, funding availability, and the long time span covered by the bibliometric corpus. Nevertheless, they suggest that factors beyond contemporary epidemiological burden contribute substantially to the observed distribution of HBV research in Peru.

This territorial imbalance carries important implications for HBV elimination strategies. The World Health Organization has emphasized that elimination targets require locally adapted epidemiological evidence capable of guiding interventions in high-risk and underserved populations [1]. Yet the predominance of locally scoped studies combined with the underrepresentation of endemic departments suggests that substantial portions of the Peruvian HBV burden may remain poorly characterized in the indexed literature. Similar concerns have been raised in other LMIC settings, where insufficient regional research capacity constrains surveillance quality and delays implementation of evidence-based interventions [27,28,29]. Our findings therefore suggest that an important challenge for Peru may be the limited geographic alignment between research activity and epidemiological need, although this interpretation should be considered in light of historical endemicity patterns, institutional capacity, and funding availability.

Thematic analysis revealed an equally important structural limitation. Peruvian HBV research has historically remained anchored in descriptive epidemiology, seroprevalence studies, blood donor screening, and vaccination coverage assessments [9,10,30,31]. These themes occupied the most central and cohesive positions in both the strategic thematic map and the co-occurrence network, whereas molecular virology, therapeutic research, and basic sciences remained peripheral or weakly connected. This contrasts sharply with the current international HBV research landscape, which increasingly focuses on functional cure strategies, host immune modulation, cccDNA biology, antiviral resistance, and precision molecular surveillance [32,33,34,35,36,37,38]. Although recent Peruvian contributions have begun to address more advanced molecular topics, including HBV genomics and evolutionary analyses covering mutations associated with occult HBV infection (OBI) and antiviral resistance [6], these studies remain relatively infrequent within the overall national research landscape. Consequently, the thematic profile of Peruvian HBV research continues to be dominated by epidemiological and seroprevalence-oriented investigations, while molecular and translational approaches remain comparatively underrepresented. Recent global reviews have emphasized that HBV research priorities are progressively shifting toward translational and therapeutic innovation, including novel immunotherapeutic approaches and biomarkers predictive of functional cure [32,33,34,35,38]. In contrast, our Sankey analysis demonstrated that no thematic lineage associated with therapeutic intervention emerged consistently across the three historical periods analyzed. This persistent discontinuity is particularly concerning, as it suggests the absence of a sustained and cumulative research agenda capable of supporting the development of long-term evidence-based therapeutic strategies for HBV management in Peru.

Recent international HBV research has progressively expanded beyond descriptive epidemiology and prevalence studies toward areas such as functional cure strategies, host–virus interactions, cccDNA biology, antiviral resistance, molecular surveillance, and the development of novel therapeutic approaches [39,40,41,42]. These topics are increasingly recognized as major priorities in the global HBV research agenda because of their potential to improve disease control and support hepatitis elimination goals [1]. In contrast, the thematic structure identified in the present study remained predominantly focused on epidemiological surveillance, seroprevalence, and prevention-related research, with comparatively limited representation of molecular, translational, and therapeutic investigations. Although these differences should be interpreted cautiously, they suggest opportunities to strengthen the integration of Peruvian HBV research with emerging international priorities.

The limited integration of molecular and therapeutic themes into the Peruvian research network likely reflects broader structural constraints affecting biomedical science in resource-limited settings. Molecular virology research requires sustained access to sequencing infrastructure, high-level biosafety laboratories, bioinformatics training, and long-term competitive funding—resources that remain unevenly distributed throughout Peru. Franzen et al. [43] highlighted that many LMIC research systems remain trapped in cycles of short-term project funding and weak institutional continuity, preventing transition from descriptive public-health research toward technologically intensive biomedical science. Similarly, Ghaffar et al. [44] argued that sustainable health-system strengthening depends on embedding research capacity directly within national health infrastructures rather than concentrating expertise in isolated academic centers. The concentration of Peruvian HBV research within a small number of Lima-based institutions likely reflects this broader systemic dynamic.

Nevertheless, the predominance of epidemiological and vaccination-related studies should not be interpreted solely as a weakness. Peru historically represented one of the most important hyperendemic HBV settings in South America, particularly within Amazonian indigenous communities and selected Andean populations [24,7]. Consequently, early research priorities understandably focused on documenting prevalence, transmission patterns, and vaccine implementation. The emergence of vaccination and prevention clusters during the second thematic period coincides temporally with the introduction of targeted immunization strategies in Amazonian populations during the 1990s [45] and the later expansion toward universal infant vaccination in 2003 [9,46]. These transitions illustrate how national public-health policies directly shaped scientific production. Similar bibliometric shifts have been described in other infectious disease fields, where implementation of vaccination campaigns or national control programs stimulated corresponding increases in epidemiological and surveillance-oriented publications [47,48].

The co-authorship and institutional analyses additionally revealed a fragmented collaborative ecosystem characterized by limited internationalization. Only 10.3% of studies involved international collaboration, a proportion substantially lower than that reported for many other biomedical research fields in Latin America [21,49,50,51]. International partnerships were dominated by the U.S. Naval Medical Research Unit No. 6 (NAMRU-6), which has historically contributed to infectious disease surveillance and molecular epidemiology research in Peru [52,53]. Although collaborations with the United States, Brazil, and several European countries became more visible during recent years, the absence of partnerships with many highly endemic regions worldwide—particularly sub-Saharan Africa and East Asia—is notable [6,10,54,55,56,57]. International collaboration is widely recognized as a key determinant of scientific visibility, citation impact, and technological transfer, particularly in LMIC settings [49,58,59]. The comparatively weak collaborative integration observed here may therefore contribute to the thematic stagnation and limited translational progression of the national HBV research agenda. The limited international collaboration observed in the present study is consistent with broader patterns reported across several Peruvian and Latin American research systems. A recent bibliometric analysis of Microbiology and Parasitology research documented an even lower rate of genuine international collaboration (1.8%), together with a marked concentration of scientific production in Lima-based institutions, suggesting that restricted international integration may represent a persistent structural characteristic of the Peruvian research landscape rather than a phenomenon specific to HBV research [20]. Similar challenges have been described in other low- and middle-income countries (LMICs), where scientific production often remains concentrated within a limited number of institutions and research hubs despite sustained growth in publication output [60,61,62]. Previous studies have shown that international collaboration facilitates access to advanced infrastructure, specialized expertise, research funding, and greater scientific visibility, all of which contribute to higher-impact research outputs [63,64,65]. In this context, the relatively low level of international collaboration identified in Peruvian HBV research suggests opportunities to strengthen integration into global research networks and accelerate the development of more diverse and translational research agendas.

The authorship distribution further demonstrated a weakly consolidated scientific elite. Although a small group of researchers accounted for a disproportionate share of publications, the Price elite threshold was not reached, suggesting the absence of a mature and highly interconnected national research core [66]. This pattern contrasts with more consolidated infectious disease research systems, where stable collaborative networks facilitate continuity, mentorship, and cumulative expertise. The predominance of single-publication authors in our corpus may reflect the large contribution of undergraduate theses and transient academic projects rather than sustained career-oriented research trajectories. Similar productivity fragmentation has been described in other emerging scientific systems and is frequently associated with discontinuous funding and limited institutional support for early-career investigators [67,68,69,70].

Importantly, this study also demonstrates the value of integrating thesis into bibliometric analyses conducted in LMIC contexts. Conventional bibliometric studies relying exclusively on indexed databases frequently underestimate national scientific production in countries where theses, institutional repositories, and regional journals constitute substantial components of the evidence ecosystem. By incorporating RENATI alongside Scopus and Google Scholar, our study captured a much broader representation of Peruvian HBV research activity. Previous methodological reviews have emphasized that exclusion of gray literature may distort thematic and geographic analyses, particularly in countries with heterogeneous indexing practices [71,72]. Although gray literature may present variable methodological quality, its inclusion is essential for accurately characterizing national research landscapes in resource-constrained settings.

The present study contains some limitations that should be acknowledged. First, historical under-digitization of older Peruvian biomedical literature may have contributed to incomplete retrieval of pre-1988 publications. Second, the title-restricted Google Scholar search strategy, adopted to maximize thematic specificity, may have excluded potentially relevant studies in which hepatitis B was an important component of the investigation but was not explicitly mentioned in the title. Third, the burden analysis was based on contemporary incidence estimates, whereas the bibliometric corpus spans several decades; therefore, temporal changes in endemicity may not be fully captured by this comparison. Finally, citation-based metrics were not incorporated because standardized citation metadata were unavailable across all included sources, particularly within RENATI. Despite these limitations, the combined multi-source strategy and reproducible analytical framework substantially strengthen the comprehensiveness of the present study.

Taken together, our findings indicate that Peru has achieved quantitative growth in HBV research without proportional diversification, decentralization, or integration into the global HBV innovation frontier. Achieving the WHO 2030 elimination goals will require structural reforms extending beyond surveillance expansion alone. Competitive funding mechanisms should incorporate explicit geographic-equity criteria prioritizing endemic regions, while long-term investments in regional sequencing infrastructure, bioinformatics capacity, and translational virology programs are urgently needed. The bibliometric inequities documented here likely extend beyond HBV and may characterize multiple endemic infectious diseases across Peru and other Latin American countries. Consequently, the analytical framework developed in this study may serve as a scalable model for evaluating research equity and thematic alignment in other neglected or geographically concentrated diseases.

## 5. Conclusions

This study provides the first comprehensive bibliometric overview of hepatitis B research in Peru and indicates that, despite sustained growth in scientific production over recent decades, important structural inequities persist across geographic, institutional, and thematic dimensions. National output remains concentrated in a limited number of Lima-based institutions, while several regions with documented HBV endemicity remain comparatively underrepresented within the scientific literature. In parallel, the thematic landscape remains dominated by descriptive epidemiology and vaccination-related studies, with comparatively limited development of molecular virology, translational science, and therapeutic research.

These findings have direct implications for the achievement of the WHO 2030 viral hepatitis elimination targets in Peru and comparable LMIC settings. Future progress will require not only increased publication volume, but also strategic decentralization of research capacity, sustained investment in regional scientific infrastructure, stronger international collaboration, and integration of molecular and clinical research approaches into national HBV agendas. Although departmental scientific production was not significantly associated with contemporary HBV incidence, the observed geographic concentration of research activity suggests opportunities to strengthen research capacity in historically affected regions. Beyond HBV, the bibliometric framework presented here may serve as a scalable model for evaluating research equity and thematic alignment in other endemic infectious diseases across Latin America.

## Data Availability

The dataset supporting this study is provided as Supplementary Tables: Bibliometric characteristics and analytical data of the included scientific publications. It contains metadata for selected articles, including journal, year, thematic area, study design, production type, institutional affiliation, geographic origin, authorship, and collaboration indicators. Data is publicly available for reproducibility. The curated bibliometric database, Supplementary Materials, and all R scripts used for data processing, statistical analyses, network construction, and figure generation are publicly available at: https://github.com/jhonpalomino02-blip/HBV-Bibliometrics-Peru/tree/main (accessed on 28 April 2026).

## Supplementary Materials

The following supporting information can be downloaded at: https://www.mdpi.com/article/10.3390/pathogens15070708/s1, Supplementary Tables: Bibliometric characteristics and analytical data of the included scientific publications. Figure S1: PRISMA flow diagram mapping the literature screening and selection process for Peruvian hepatitis B bibliometric analysis. Figure S2: Percentage and absolute distribution of document types within the analyzed Peruvian hepatitis B research corpus (*n* = 232). Figure S3: Distribution of all normalized thematic concepts identified in the Peruvian HBV research corpus. Figure S4: Global network of international scientific collaboration on Peruvian hepatitis B research (1988–2023). Figure S5: Absolute number of publications co-authored with Peru and corresponding international collaboration ratio for the top contributing countries (1988–2023).
