# Supplementary figures and images for: Hepatitis B Research in Peru, 1988–2023: Geographic Inequities, Thematic Gaps, and Misalignment with Disease Burden
Source: Pathogens. 2026 Jul 6;15(7):708. doi: 10.3390/pathogens15070708 (PMC13414854; doi:10.3390/pathogens15070708)

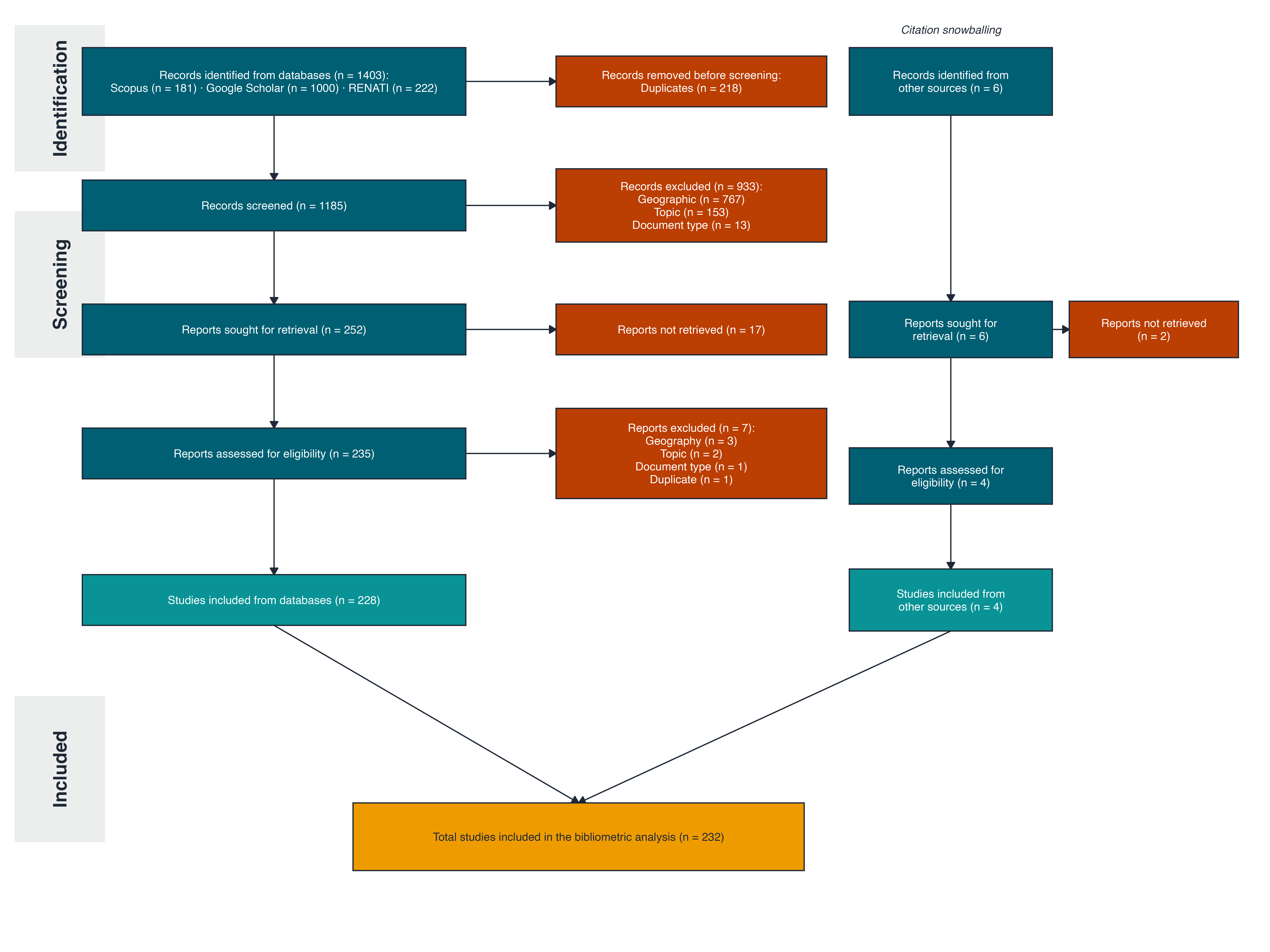

Supplement: Supplementary file 1 [file pathogens-15-00708-s001.zip › Figure S1.png]

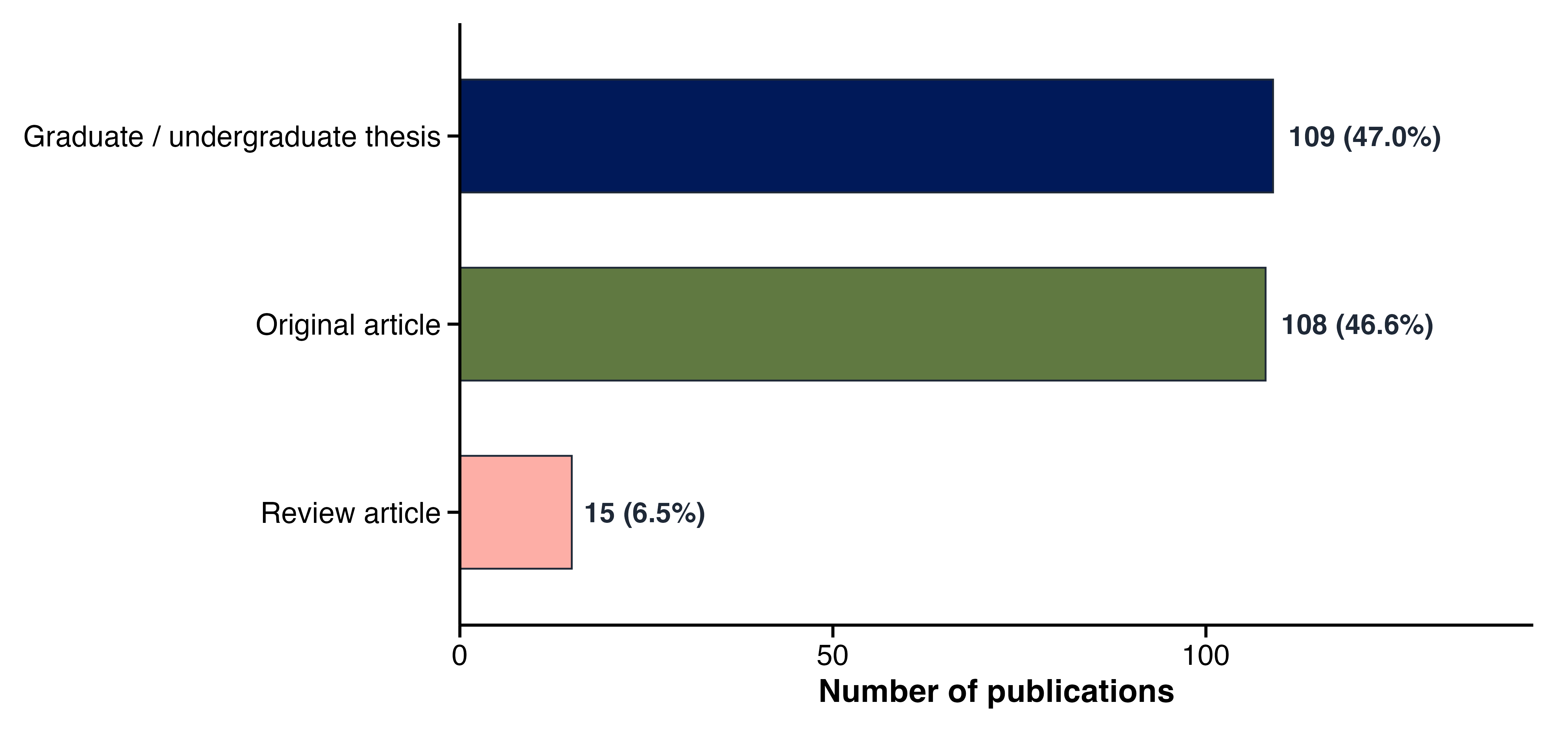

Supplement: Supplementary file 1 [file pathogens-15-00708-s001.zip › Figure S2.png]

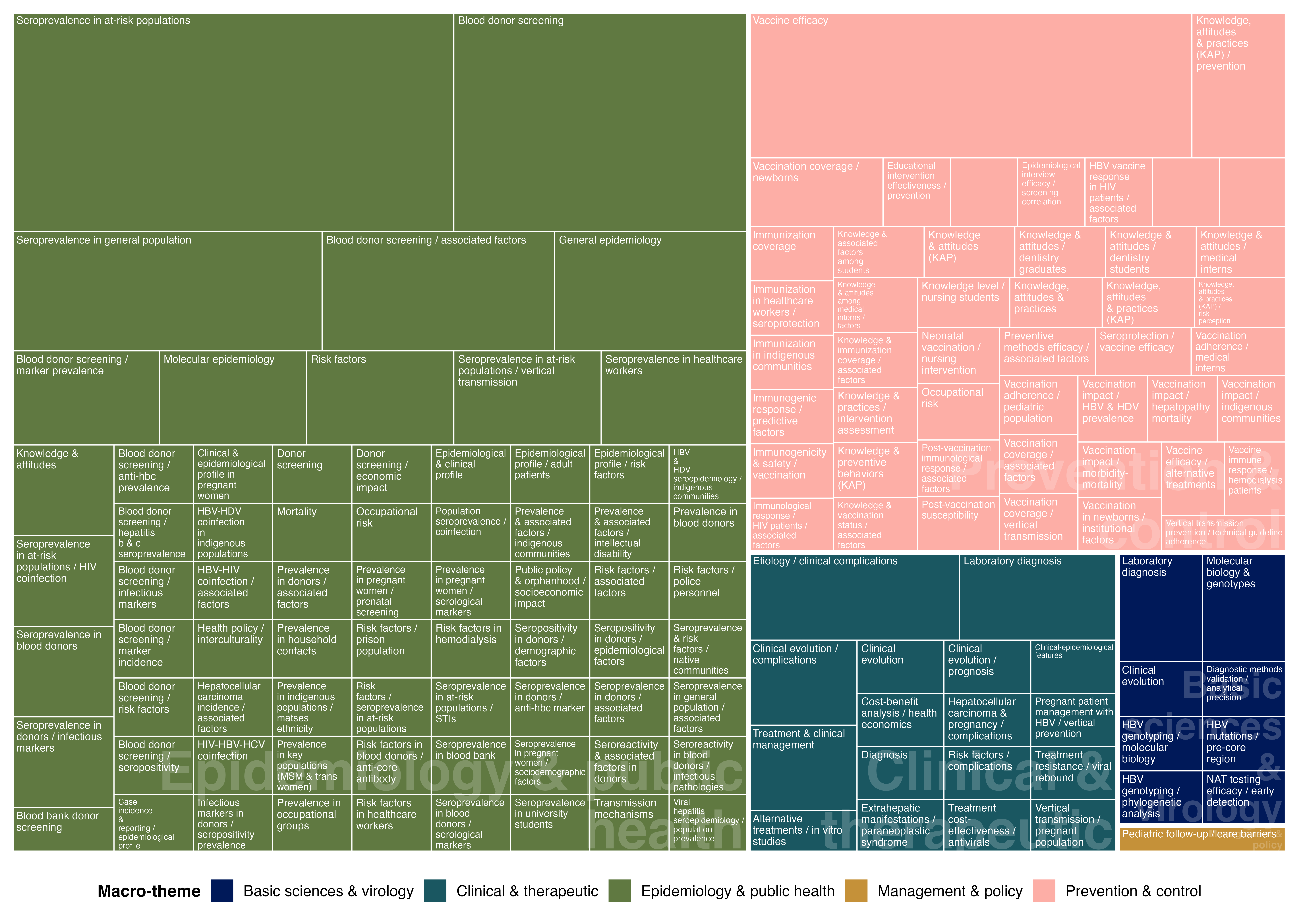

Supplement: Supplementary file 1 [file pathogens-15-00708-s001.zip › Figure S3.png]

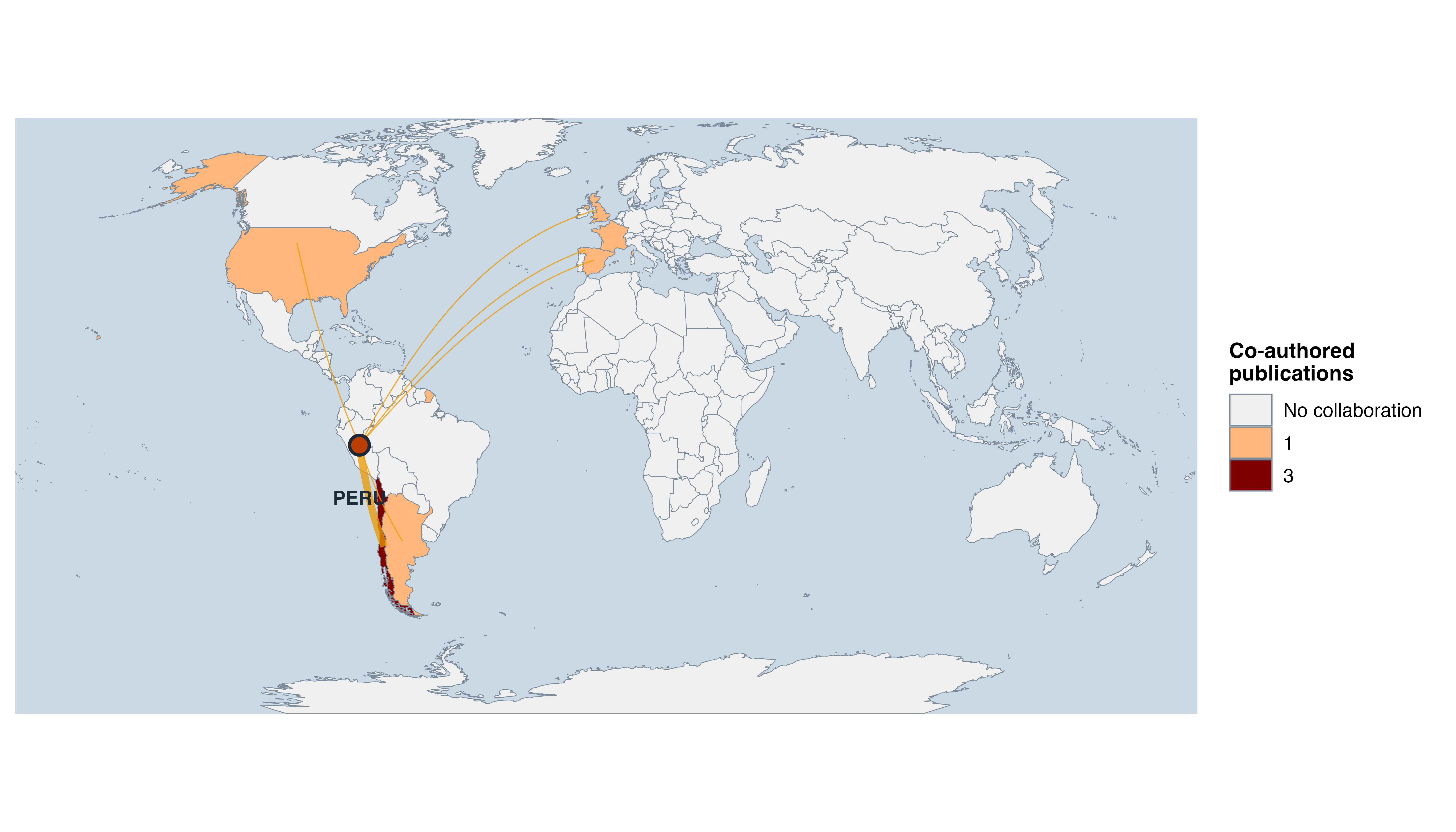

Supplement: Supplementary file 1 [file pathogens-15-00708-s001.zip › Figure S4.png]

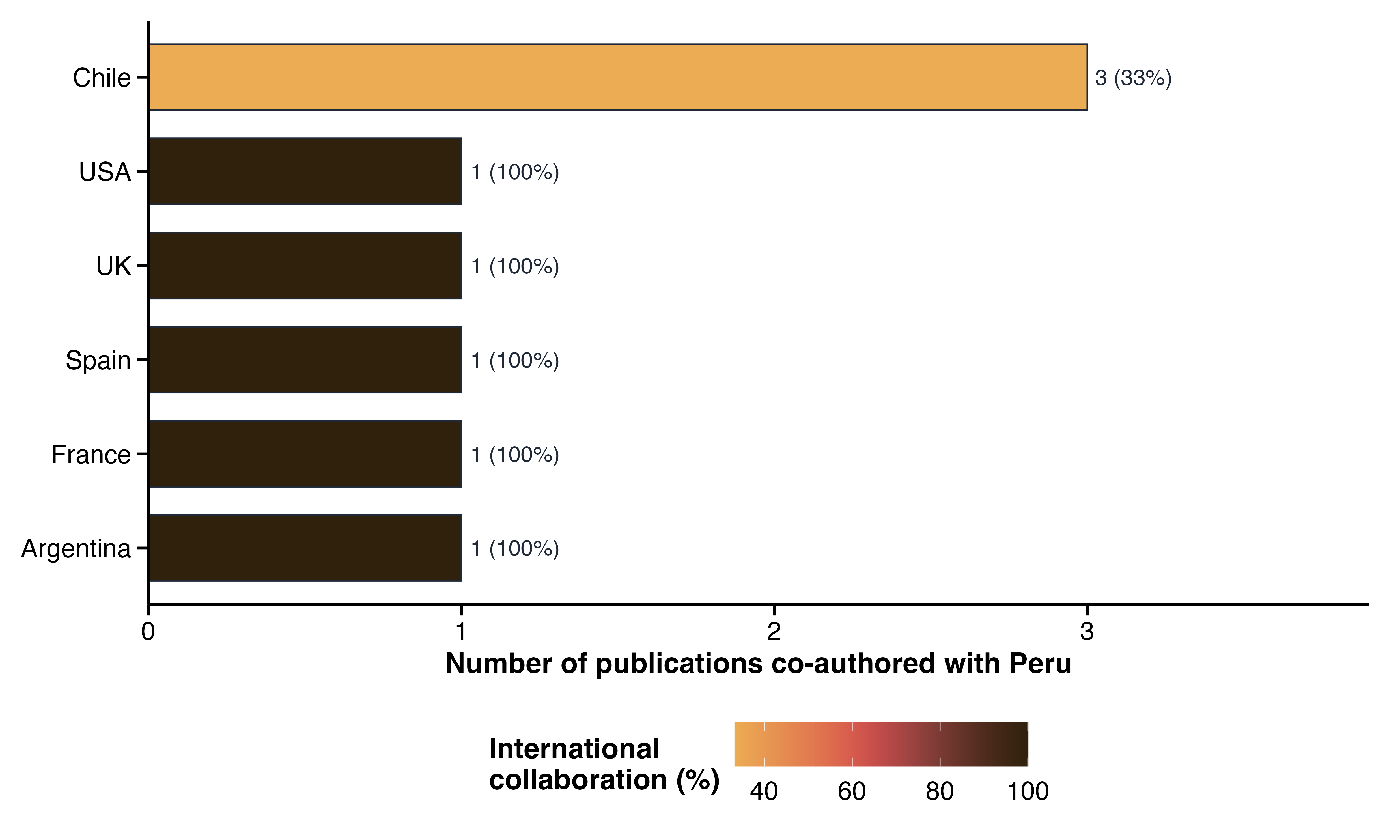

Supplement: Supplementary file 1 [file pathogens-15-00708-s001.zip › Figure S5.png]
